# Brain Clearance of Contrast Agent in Intravenous DCE‐MRI Is Measurable and Cannot Be Explained by Clearance Across the BBB Alone

**DOI:** 10.1002/mrm.70514

**Published:** 2026-07-18

**Authors:** Martin Kozár, Ioana‐Emilia Mosneag, Sarah Al‐Bachari, Igor Chernyavsky, Geoff J. M. Parker, Hervé Boutin, Laura M. Parkes, Ingo Schiessl, Ben R. Dickie

**Affiliations:** ^1^ Division of Imaging, Informatics and Data Sciences, School of Health Sciences, Faculty of Biology, Medicine and Health University of Manchester Manchester UK; ^2^ Geoffrey Jefferson Brain Research Centre, Faculty of Biology, Medicine and Health, University of Manchester, Manchester Academic Health Science Centre Manchester UK; ^3^ Department of Clinical and Movement Neurosciences University College London London UK; ^4^ Department of Mathematics, School of Natural Sciences, Faculty of Science and Engineering University of Manchester Manchester UK; ^5^ Maternal and Fetal Health Research Centre, School of Medical Sciences University of Manchester Manchester UK; ^6^ Quantitative Imaging Group, UCL Hawkes Institute, Department of Medical Physics & Biomedical Engineering University College London London UK; ^7^ Bioxydyn Limited Manchester UK; ^8^ Imaging Brain and Neuropsychiatry iBraiN 1253, Université de Tours, Inserm, Bat Planiol, UFR de Médecine Tours France; ^9^ Division of Neuroscience, School of Biological Sciences, Faculty of Biology, Medicine and Health, Manchester Academic Health Science Centre The University of Manchester Manchester UK; ^10^ Division of Psychology, Communication and Human Neuroscience, School of Health Sciences, Faculty of Biology, Medicine and Health University of Manchester Manchester UK; ^11^ Division of Pharmacy and Optometry, School of Health Sciences, Faculty of Biology, Medicine and Health University of Manchester Manchester UK

## Abstract

**Purpose:**

To determine the feasibility of measuring brain clearance of gadolinium contrast agent using standard intravenous DCE‐MRI acquisitions and evaluate the contribution of blood–brain barrier (BBB) and non‐BBB clearance routes.

**Methods:**

Uptake and extended Tofts models were fit to DCE‐MRI data from people with Parkinson's disease, post‐stroke and controls. Models were compared using the Akaike information criterion. Key parameters (*K* ^trans^, *v*_p_, *v*_e_, and *k*) were extracted from gray and white matter ROIs. In mice, two‐photon microscopy of intravenously injected Sulforhodamine 101 was used to confirm small tracer clearance kinetics without the confounds of partial volume effects or water exchange.

**Results:**

The extended Tofts model provided a superior fit compared to all uptake‐only models. Extended Tofts estimates of the extravascular extracellular volume fraction *v*_e_ were underestimated by a factor of 10–20 compared to known literature values (1.5% vs. 20%–30%). We hypothesized this bias may be due to an additional competing non‐BBB clearance mechanism not accounted for by the extended Tofts model. In both MRI and two‐photon data, we found that *v*_e_ estimates trended toward literature values as BBB permeability (and thus BBB clearance) increased, supporting our hypothesis.

**Conclusion:**

Modeling clearance of extravasated contrast agent from the brain improves fit quality compared to models that neglect clearance. Estimates of interstitial volume fraction were underestimated at low *K* ^trans^ but tended toward literature values as BBB permeability (and clearance) increased. This study indicates that estimating brain clearance of contrast agent using DCE‐MRI is feasible, and that estimates reflect a combination of BBB and non‐BBB clearance pathways.

## 1 Introduction

Dynamic contrast‐enhanced MRI (DCE‐MRI) of intravenously injected contrast agents is gaining popularity for assessment of subtle blood–brain barrier (BBB) leakage associated with neurodegeneration and aging [1, 2, 3, 4, 5]. In this context, leakage of contrast agent from the bloodstream into the brain is typically slow (*K* ^trans^ < 0.01 min^−1^) and contrast agent does not reach sufficiently high concentrations in the brain parenchyma during the imaging session to drive a net return to the bloodstream [4]. As such, clearance of extravasated contrast agent from the brain is typically ignored in modeling approaches, and the Patlak model is used for tracer kinetic analysis in normal appearing brain tissue [6].

In contrast, it has been known for decades that BBB impermeable tracers injected into the brain are cleared at a rate that does not depend on their size, highlighting the existence of a secondary advective clearance pathway complementary to the BBB [7, 8, 9, 10, 11]. Recent studies by Iliff et al. [12] discovered the role of aquaporin‐4 (AQP4) in driving bulk flows via a peri‐arterial–perivenous drainage pathway. This work has prompted many studies in both rodent and man using cerebrospinal fluid injections of MRI or fluorescently labeled tracers to measure perivascular influx and parenchymal clearance [12, 13, 14, 15, 16, 17, 18]. In rodent brain, clearance rates for tracers injected into the CSF are of the order ∼0.02 min^−1^ [12, 17]. Tracers injected directly into the brain clear with similar rates, around 0.005–0.05 min^−1^, depending on whether the tracer is BBB permeable or not, and whether the animal is awake or anesthetized [12, 15, 16, 17, 18]. In human brain, clearance data is more limited, but CSF tracer studies of Eide and Ringstad have indicated clearance is slower, presumably because of the larger brain [19].

Being able to obtain measurements of brain clearance using intravenous DCE‐MRI, rather than injecting contrast agent into CSF, would greatly increase the applicability of these methods in clinical research. Several studies have demonstrated that modeling clearance following intravenous injections does not improve fit quality relative to fitting the Patlak model; however, these studies are based on DCE‐MRI data with low temporal resolution (> 1–2 min per volume [6, 20, 21]). In this study, we re‐evaluate model fits in high temporal resolution data (7.6 s per volume) to establish whether it is feasible to estimate parenchymal clearance of contrast agent using intravenous DCE‐MRI. We compare different modeling approaches, namely those that do and do not incorporate clearance, in patients with Parkinson's disease, post‐stroke, and healthy controls. Our primary aim was to determine whether the extended Tofts model provides a better fit relative to the commonly used Patlak model. Other factors that could plausibly affect fit quality were assessed against the extended Tofts model including modeling tracer dispersion, non‐instantaneous mixing, and finite water exchange. Multi‐photon experiments in the mouse brain were conducted to provide complementary evidence of rapid small molecule clearance kinetics without the confounds of partial volume and water‐exchange effects present in DCE‐MRI measures.

## 2 Methods

### 2.1 DCE‐MRI

#### 2.1.1 Data Acquisition

Experiments involving human participants were reviewed and approved by NHS ethical approval (North West Preston Research Ethics Committee, United Kingdom). The patients/participants provided their written informed consent to participate in this study.

All quantities, processes, and models are defined in compliance with OSIPI CAPLEX [22]. Three cohorts of participants, aged between 52 and 85 years, were included in the analysis: Parkinson's disease with no history of cerebrovascular disease (*n* = 41, mean age = 66 ± 7 years, sex (F:M) 16:15), stroke patients or those with transient ischaemic attack at least 3 months but not more than 2 years prior to imaging (*n* = 15, mean age = 69 ± 7 years, sex (F:M) 4:11), and healthy aged‐matched controls with no clinical diagnosis of either Parkinsons's disease or cerebrovascular disease (*n* = 30, mean age = 69 ± 8 years, sex (F:M) 12:36). Data acquisition is described in detail in Al‐Bachari et al. [3].

In brief, the participants were scanned on one of two 3T Phillips Achieva scanners, one with 8‐channel coil and the other with a 32‐channel coil. Both scanners ran the same DCE‐MRI protocol. Prior to the dynamic scan, a *T*_1_ map was acquired using 3D cartesian *T*_1_ Fast‐Field Echo (T1 FFE) at flip angles 2°, 5°, and 10°, NEX = 8, FOV of 192 × 192 mm and matrix size of 128 × 128, giving in‐plane resolution of 1.5 × 1.5 mm; 32 contiguous axial slices of 4 mm thickness, echo time (TE) = 0.8 ms, repetition time (TR) = 2.4 ms. The DCE‐MRI scan used the same acquisition parameters and consisted of 160 images (NEX = 1) at a flip angle of 10°. The temporal resolution was 7.6 s with a total duration of 20 min. At the start of the 8th dynamic (at 53.2 s), a bolus of gadolinium‐based contrast agent (Gd‐DOTA; Dotarem, Guerbet) of concentration 0.5 mmol/mL was administered at a rate of 3 mL/s using a power injector. The volume injected was proportional to the weight of the subject (0.2 mL/kg).

In order to correct for B1 field inhomogeneities, a B1 mapping sequence was also acquired with the same voxel size and coverage as for the variable flip angle images [23]. This consisted of a pair of 3D spoiled gradient echo images with TR_1_ = 25 ms and TR_2_ = 125 ms, flip angle 60°, TE = 5 ms, acquisition time 117 s.

#### 2.1.2 Kinetic Models

To test whether modeling contrast agent clearance improved fit accuracy, the Patlak and extended Tofts models were fit and their respective fit qualities compared using the Akaike Information Criterion (AIC). To rule out other potential factors that could improve fit quality other than clearance, we also compared the extended Tofts model to other uptake models, including the two‐compartment uptake model [24] (to assess effects of finite vascular dispersion with instantaneous mixing), the plug flow uptake model (to assess effects of dispersion with non‐instantaneous mixing) [25], and to a Patlak model with finite blood‐brain barrier water exchange (to assess the effects of finite vascular water exchange) [26]. The effects of signal drift on model selection were also assessed—methodological detail is provided later.

**FIGURE 1:**
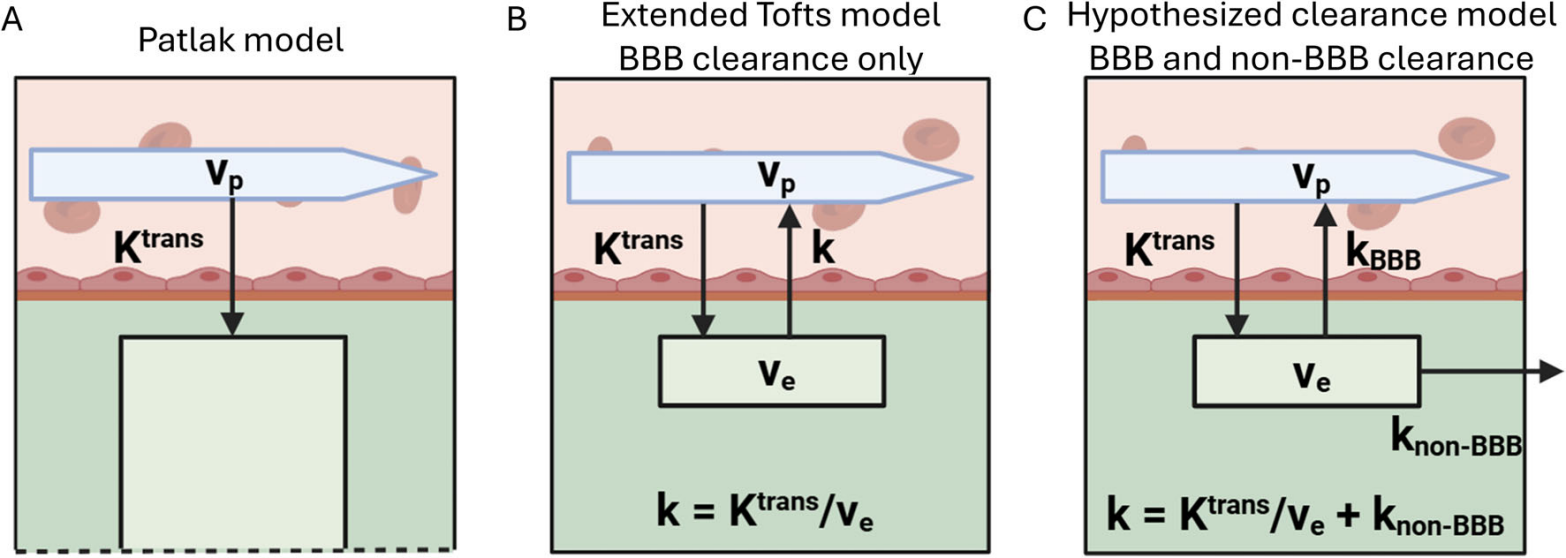
A schematic representation of the Patlak (A), extended Tofts (B) and hypothesized clearance models (C). Each diagram is split into a blood (red) and interstitial (green) compartment. The Patlak model parameters are the plasma volume fraction, *v*_p_, and the volume transfer constant, *K* ^trans^. Here, the contrast agent is modeled as crossing the BBB unidirectionally. The extended Tofts model extends the Patlak model by modeling the tracer returning to blood at a rate dependent on *K* ^trans^ and the interstitial volume fraction, *v*_e_. The hypothesized clearance model assumes clearance of contrast agent can occur through BBB and non‐BBB pathways. Mathematically, the extended Tofts and hypothesized clearance models can be described by the same general equations. The difference lies in how the clearance rate *k* is interpreted.

Figure 1 shows compartment diagrams for the Patlak, extended Tofts and general “hypothesized” clearance models. The Patlak model (Figure 1A) is defined as follows:

(1) $$C\left(t\right)=v_{P}C_{P}\left(t\right)+K^{\text{trans}}\int_{0}^{t}C_{P}\left(\tau \right)d\tau$$

where *C* [mM] is the tissue contrast agent concentration, *v*_p_ [mL mL^−1^] is the plasma volume fraction, *C*_p_ [mM] is the plasma contrast agent concentration at a feeding artery, *K* ^trans^ [min^−1^] is the contrast agent volume transfer constant, and *t* [min] is the time.

The extended Tofts model (Figure 1B,C) is a simplified case of a more general clearance model given below:

(2) $$\begin{array}{c} C\left(t\right)=v_{P}C_{P}\left(t\right)+K^{\text{trans}}\int_{0}^{t}C_{P}\left(\tau \right)e^{-k\left(t-\tau \right)}d\tau \end{array}$$

where *k* [min^−1^] is the total contrast agent clearance rate from the voxel or ROI. The extended Tofts model assumes that clearance occurs only across the BBB, such that $k=\frac{K^{\text{trans}}}{v_{e}}$, where *v*_e_ is the interstitial volume fraction. In the brain it is known that tracers can also clear via non‐BBB routes, in which case *k* can be written more generally as a combination of BBB and non‐BBB rate constants: $k=\frac{K^{\text{trans}}}{v_{e}}+k_{\text{non}-\mathrm{BBB}}$.

If *k*_non‐BBB_ is finite, *v*_e_ estimated using the extended Tofts model (which assumes $k_{\text{non}-\mathrm{BBB}}=0$) will be underestimated, with the degree of underestimation dependent on *K* ^trans^ and *k*. With knowledge of the true *v*_e_ value, the BBB and non‐BBB contributions to *k* can be estimated. In this study, we do not attempt to fit this more general model, but instead derive indicative estimates of $k_{\mathrm{BBB}}$ and $k_{\text{non}-\mathrm{BBB}}$ using measured *K* ^trans^ values and a literature value of *v*_e_ = 0.25 [27, 28, 29].

To avoid errors caused by inflow effects, *C*_p_ [mM] is measured at the sagittal sinus under the assumption that dispersion and loss of contrast agent during vascular transport are negligible.

The two compartment uptake and plug flow uptake models, which relax the zero‐dispersion assumption, are described elsewhere [24, 25].

#### 2.1.3 Data Extraction and Model Fitting

Gray matter (GM) and white matter (WM) regions of interest (ROIs) were segmented on a co‐registered structural 3D T1‐weighted image in SPM12 using a probability threshold of 95%. The average size of these GM and WM ROIs were 675 cm^3^ and 455 cm^3^ respectively. The dynamic images were corrected for motion by alignment to the first image using SPM12's realignment option. A vascular input function (VIF) was obtained from approximately 50 voxels manually segmented from the sagittal sinus in the final motion‐corrected dynamic image. For each GM or WM ROI, we calculated the mean raw MRI signal at each time‐point, producing a signal time‐course per ROI for further analysis. Differences in bolus arrival time between tissue and VIF curves was corrected prior to model fitting by shifting the tissue signal via interpolation. Signal curves were then converted to concentration timecourses for model fitting using estimates of pre‐contrast *T*_1_ and the known Gd‐DOTA relaxivity of 3.4 s^−1^ mM^−1^. All models were fit using the least squares method implemented in Python (version 3.8.19) using the scipy module (version 1.10.1). All fits were repeated 20 times by varying the starting K^trans^ value from 0.0001 to 0.005 min^−1^ to avoid local minima, choosing the fit with minimal sum of squared residuals. The bounds on *v*_p_ were set between 0 and 1 mL mL^−1^, bounds on *K* ^trans^ were set between −inf and +inf min^−1^, and bounds on *k* were set to −inf to +inf min^−1^. For dispersion models, an additional parameter denoted as the venous transit time was estimated to account for dispersion of the vascular input by the venous vasculature.

Patlak modeling is usually applied to data after the peak to exclude effects of finite tissue perfusion and delay/dispersion of the bolus [6, 30]. Model fitting and comparisons were therefore repeated multiple times excluding the first 100, 160, 220, and 280 s of data to assess the impact of eliminating progressively more data after the peak. The peak in the VIF occurred between ∼68 and 76 s. The null hypothesis of no difference in AIC between the extended Tofts and Patlak model at each timepoint was tested using an ANOVA with post hoc *t*‐tests.

Two compartment uptake and plug flow models were fit in a two‐step approach: (i) first the venous VIF was deconvolved with venous and capillary impulse response functions to generate an arterial input function, (ii) the estimated arterial input function was applied in combination with the two compartment uptake model (modeling dispersion with instantaneous mixing) [24] and plug flow uptake model (modeling dispersion with non‐instantaneous mixing) [25]. The venous impulse response function was modeled as an exponential with mean transit time *T*_v_. The capillary impulse response functions for the two‐compartment uptake model and plug flow uptake models are defined here [24, 25].

Finite BBB water exchange was also assessed. Here, the Patlak model was fit to raw signal intensities assuming a biexponential model for water exchange effects on blood and tissue *R*_1_ [31]. The blood water residence time was fixed at *τ*_b_ = 0.377 s, which is an average of literature values taken from Dickie et al. [26]. The standard extended Tofts model was also fit in the signal domain for comparison with the Patlak finite exchange model.

#### 2.1.4 Model Comparison

The goodness‐of‐fit of each model was determined using Akaike information criterion (AIC) [32], which penalizes models with more parameters *p*. The AIC value for a model *m* is given by

(3) $$\begin{array}{c} {\mathrm{AIC}}_{m}=N\cdot \ln\left(\frac{\mathrm{SSE}}{N}\right)+2\left(p+1\right) \end{array}$$

where *N* is the number of observations, SSE is the sum of squared errors between the model and data, and *p* is the number of model parameters.

The Patlak model has two parameters (*K* ^trans^ and *v*_p_), the extended Tofts (*K* ^trans^, *v*_p_, *v*_e_) and finite exchange Patlak model (*K* ^trans^, *v*_p_, *τ*_b_) have three parameters, the two compartment uptake model (*K* ^trans^, *v*_p_, *T*_b_, *T*_v_) has four parameters, and plug flow uptake model (*K* ^trans^, *v*_p_, *T*_b_, *T*_l_, *T*_v_) has five parameters. *K* ^trans^, *v*_p_, *v*_e_ are defined above. *T*_b_ [s] and *T*_v_ [s] are the mean transit time of blood in capillary and venous compartments. *τ*_b_ [s^−1^] is the mean residence time of blood water in the capillary compartment prior to exchange with tissue water. *T*_l_ [s] is the capillary leakage time.

The AIC difference between candidate uptake models (e.g., Patlak, two compartment uptake etc.) and the extended Tofts model was computed using *t* = 100 s cutoff and the null hypothesis of ΔAIC = 0 tested using ANOVA with post hoc *t*‐tests.

#### 2.1.5 Temporal Resolution

Effects of temporal resolution on our ability to distinguish model fits was tested by downsampling the acquired tissue curves and VIFs to the same temporal resolution as Heye et al. [6] (Δ*t* = 73 s). Patlak and extended Tofts models were fit to the downsampled data and the difference in AIC was calculated. An ANOVA with post hoc *t*‐tests was performed to test the null hypothesis of ΔAIC = 0.

#### 2.1.6 Scanner Drift

Scanner instabilities can cause signal drift, which could mimic clearance of contrast agent from the brain. To assess scanner drift within our data, we fit a linear model to the baseline GM and WM tissue concentrations. To test whether the observed level of drift could mimic clearance, we simulated Patlak model concentration time‐courses with differing levels of concentration drift representative of the measured drift. Both linear and exponential drift functions were simulated. Values for *K* ^trans^ and *v*_p_ were set to 0.0005 min^−1^ and 0.03 mL mL^−1^. Zero mean gaussian noise was added with standard deviation of 0.001 mM. The VIF was derived from a mean of all control participants. The synthetic curves with drift were fit using Patlak and extended Tofts models and the AIC difference calculated.

### 2.2 Intravital Two‐Photon Microscopy

#### 2.2.1 Data Acquisition

Dynamic intravital two‐photon microscopy was used to determine the leakage and clearance of a small molecular weight tracer of similar size to Gd‐DOTA at the cellular level. This allowed direct evaluation of tracer leakage and clearance without the confounds of partial volume and water‐exchange effects present in DCE‐MRI experiments.

Male C57BL/6 mice (Charles River UK Limited, Kent, UK) in three age groups were used: young (*n* = 2, 17–22 weeks, 27–36 g), middle‐aged (*n* = 2, 55–59 weeks, 35–37 g) and old (*n* = 3, 87 weeks, 40–57 g). The mice were given access to food and water ad libitum and housed in groups of 2–3 at a constant ambient temperature of 21°C ± 2°C and humidity of 40%–50%, on a 12 h light, 12 h dark cycle. Animal experiments were approved by the University of Manchester's biological services facility and were conducted according to the Animals (Scientific Procedures) Act 1986 (ASPA) under relevant project license (PPL) authorisation.

The mice underwent surgery under 2% isoflurane to implant a head plate and cranial window 1 week prior to imaging over the left somatosensory cortex. The surgical procedure maintains the dura, ensuring the brain pressures remain physiological. The details of the cranial window implantation were according to the guidelines published in Gray et al. [33]. Following surgery, the mice were monitored for general health as well as changes to weight for 2 days. All mice recovered from the surgery without complications, with a maximum weight loss of 7.6% of pre‐surgery weight.

Intravital two‐photon imaging was done on a Leica DM6 B upright microscope using the HC FLUOTAR L 25×/0.95 W VISIR objective controlled by LAS X 3.5.7 software. The mice were anesthetized using 5% isoflurane for induction and maintained at 2% isoflurane in 100% O_2_ during imaging. A stereotaxic frame was used for head fixation during imaging. Body temperature was maintained constant at (37.5°C ± 0.5°C) using a heat pad. The mice received intravenous injections of two fluorescent tracers: FITC‐dextran (weight 10 000 Da, emission peak at 524 nm) was injected first as an intravascular tracer to visualize blood vessels and Sulforhodamine 101 (SR101, molecular weight 606.7 Da, emission peak at 593 nm) to mimic and assess leakage and clearance properties similar to those of Gd‐DOTA [34]. Following the dextran injection, an exploratory image of the entire cranial window was taken to locate candidate regions for scanning (see Figure S1). Large arteries (vessels > 100 μm in diameter) were excluded from the field of view.

The depth of imaging below the cortical surface was chosen by localizing the dura via second harmonics imaging. The posterior side of the dura was determined to be at the location where dura collagen fibers, presented in yellow, disappear. The dynamic scan was set to 60–100 μm below this point. After setting the dynamic imaging plane, 50 μL of SR101 was injected via the tail vein (5 mg/mL in PBS, Sigma Aldrich). The dynamic scan was set to begin 2 min following injection to prevent detector overexposure. Acquisition parameters were: matrix size = 1024 × 1024, 6 slices, FOV = 345 × 345 × 20 μm, temporal resolution 16.26 s, 74 dynamics for a total of 20 min.

#### 2.2.2 Kinetic Modeling

A maximum intensity projection was used to collapse the 4D (x, y, z, t) two‐photon image stack along the z‐direction into a 3D stack (x, y, t). The stack was corrected for inter‐frame motion by estimating x–y translations using the pyStackReg package [35]. To improve the quality of alignment, translation parameters were calculated on a normalized and denoised image stack (normalized to 0–255 and denoised using the BM3D algorithm [36, 37]), then translations were applied to the raw data.

To estimate leakage and clearance rates of SR101, the extended Tofts model was fit to dynamic SR‐101 data. The signal intensity of SR‐101 was assumed proportional to tracer concentration. The two‐photon VIF was selected by taking the average signal in an ROI drawn manually within vessels (white area in Figure 4B). The extravascular SR101 signal was obtained by dilating the vessel ROI then subtracting this from a whole image mask (light gray area in Figure 4B). Since we were able to directly measure the extravascular concentration, we did not model a separate *v*_p_ *C*_p_ contribution required to account for partial volume effects in DCE‐MRI. Both the VIF and extravascular SR101 signal were extrapolated to *t* = 0 assuming an exponential decay. The extended Tofts model was fit to each mouse dataset to estimate *K* ^trans^, *k*, and *v*_e_.

## 3 Results

**FIGURE 2:**
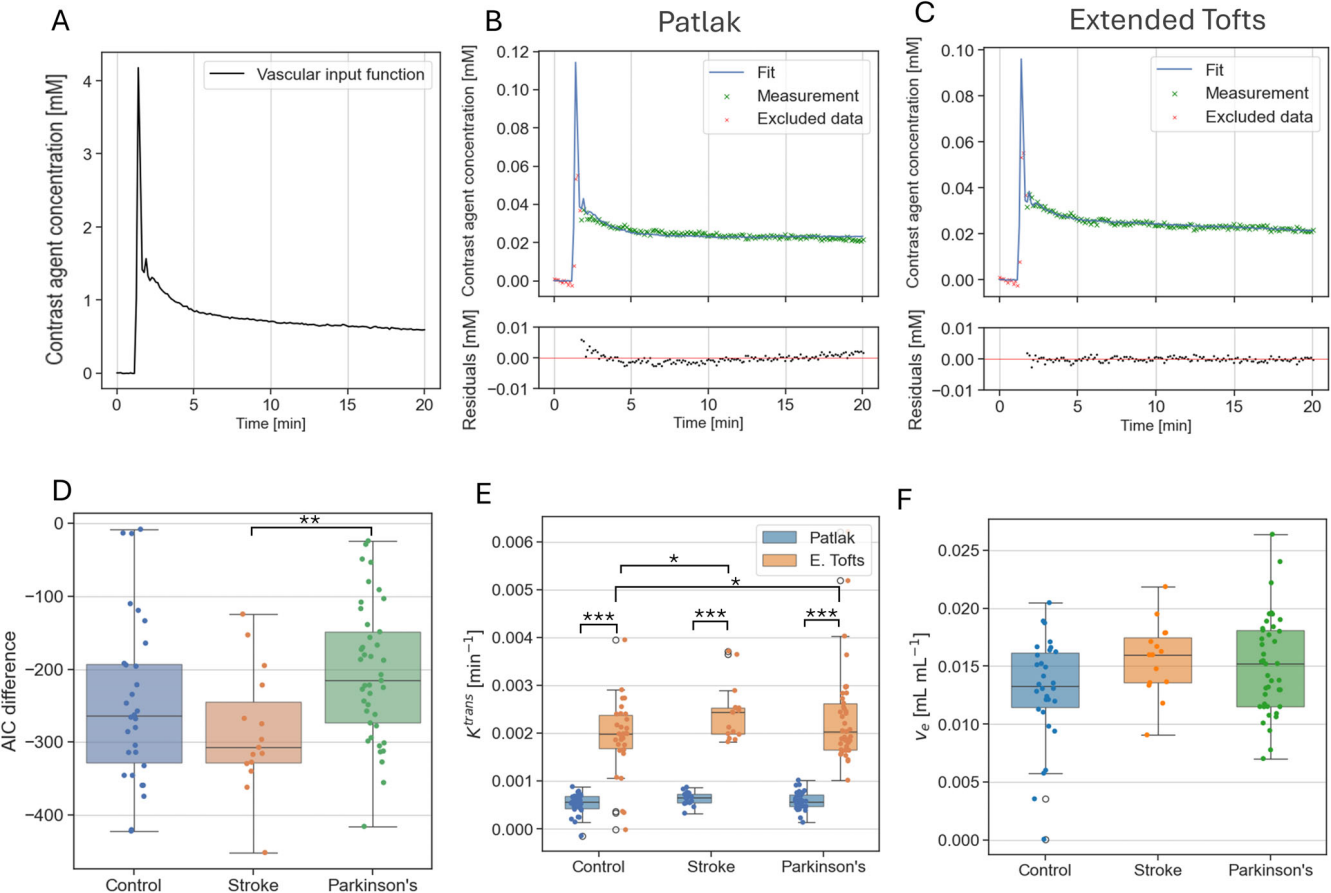
Results of DCE‐MRI kinetic modeling in gray matter of the human brain. (A) Time course of the vascular input function obtained from superior sagittal sinus. (B, C) Example plots of Patlak (Part B) and extended Tofts (Part C) fits to an example healthy control subject after removing the first 100 s of data. (D) The difference in Akaike information criterion values between Patlak and extended Tofts models in gray matter for the three patient categories. (E) Estimates of *K* ^trans^ using the Patlak and extended Tofts model for the three patient categories. (F) The estimates of the interstitial volume fraction *v*_e_ using the extended Tofts model for the three patient categories. *p < 0.01; **p < 0.001; ***p < 0.0001.

Figure 2A–D shows an example DCE‐MRI VIF, GM tissue concentration time curves and model fits for a representative control subject, and GM AIC differences (Extended Tofts minus Patlak) for the three groups of subjects.

The Patlak model overestimated Gd‐DOTA tissue concentrations near the vascular peak, underestimated concentrations between 4 and 12 min, then overestimated again after ∼12 min. The extended Tofts model provided an improved fit at all timepoints, which is highlighted by significantly lower AIC in Figure 2D. To rule out poor equilibration of contrast agent affecting AIC results, the fitting was repeated by excluding increasing amounts of data (Figure S2). The same data for WM is presented in Figure S3. In WM, AIC differences between the Patlak model and extended Tofts model were much smaller than for GM.

Figure 2E shows DCE‐MRI *K* ^trans^ in GM for Patlak and extended Tofts models. *K* ^trans^ for Patlak was within the range of previously reported values [6]. *K* ^trans^ for extended Tofts was approximately 4 times larger than Patlak *K* ^trans^. There was a significant difference in *K* ^trans^ between Patlak and extended Tofts models (*p* < 0.0001). Furthermore, we found a significant difference in GM *K* ^trans^ between Controls and Stroke, and Controls and Parkinson's for the extended Tofts model (*p* < 0.05), but not the Patlak model.

Figure 2F shows *v*_e_ estimates, which are much lower than expected based on known literature values for the extravascular extracellular space volume fraction (1.5% vs. ∼25%, [38]). There was no difference in *v*_e_ between patient groups.

**FIGURE 3:**
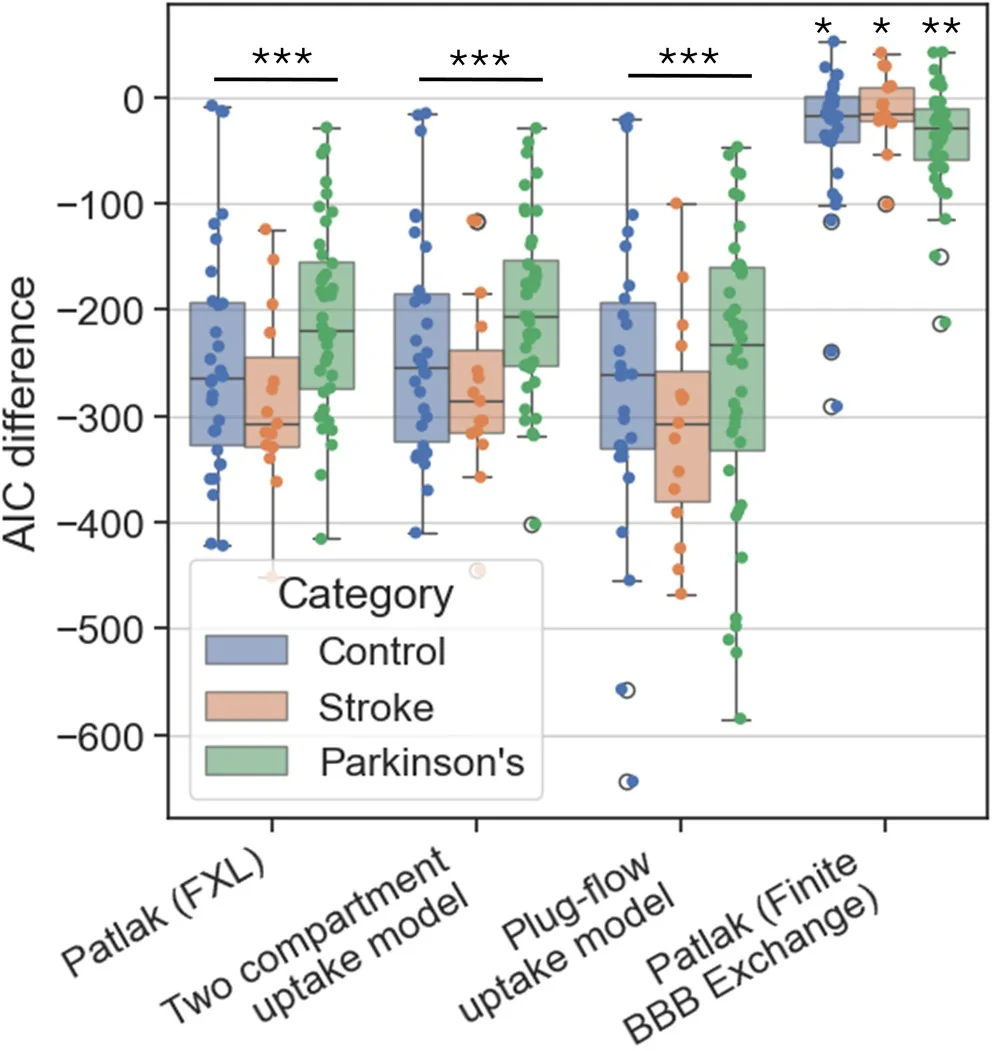
Plots showing the Akaike information criterion (AIC) difference between the extended Tofts model and candidate uptake models. AICs were calculated excluding the first 100 s of data from fits. The Patlak FXL (fast exchange limit) is a replot of Figure 2D to aid comparison with other models. The two‐compartment uptake model and plug flow uptake models led to no reduction in the AIC difference, indicating that dispersion and non‐instantaneous mixing of the contrast agent bolus in the capillary bed does not impact fit quality. A comparison of extended Tofts model to the Patlak model with finite BBB water exchange led to the greatest reduction in AIC difference. In a small number of cases, the finite water exchange model provided a better fit than extended Tofts, however, on average extended Tofts model was still superior (all ΔAIC < 15, *p* < 0.01). Stars denote the significance of post hoc *t*‐tests for each box plot testing the null hypothesis that ΔAIC = 0. *p < 0.01; **p < 0.001; ***p < 0.0001.

Figure 3 shows the AIC differences between extended Tofts model and other candidate models, including the two‐compartment uptake model, plug uptake model, and Patlak model with finite BBB water exchange. The Patlak model with fast BBB water exchange is replotted for comparison with the other models. For all model comparisons, the extended Tofts model was found to produce a significantly lower AIC (i.e., ΔAIC < −15). Modeling finite BBB water exchange improved fit quality compared to assuming the fast exchange limit, but extended Tofts still led to lower AIC.

Figure S4 shows the AIC differences between Patlak and extended Tofts model following temporal down‐sampling to match the resolution of Heye et al. [6]. It can be observed that AIC differences are smaller than at high temporal resolution, showing that higher sampling rate increases our ability to distinguish Patlak from extended Tofts. The extended Tofts model was still superior to Patlak for all patient groups in GM (*p* < 0.0001), but was only marginally better in Parkinsons's WM (*p* = 0.044) and no better for control or stroke WM (*p* > 0.05).

Figure S5A shows drift estimates obtained by fitting a linear model to the baseline of control, Parkinson's and stroke datasets. The drift estimates ranged from −1 to +1 × 10^−6^ mM/s. Assuming linear drift for the entire scan duration, the mean absolute drift measured in GM is equivalent to a change of 1 μM over 20 min. To determine if these levels of drift could mimic clearance of contrast agent, linear and exponential drifts of the same order were simulated (Figure S5B,C). In both cases, the extended Tofts model did not perform better than the Patlak, indicating that signal drift of the type simulated does not mimic contrast agent clearance.

**FIGURE 4:**
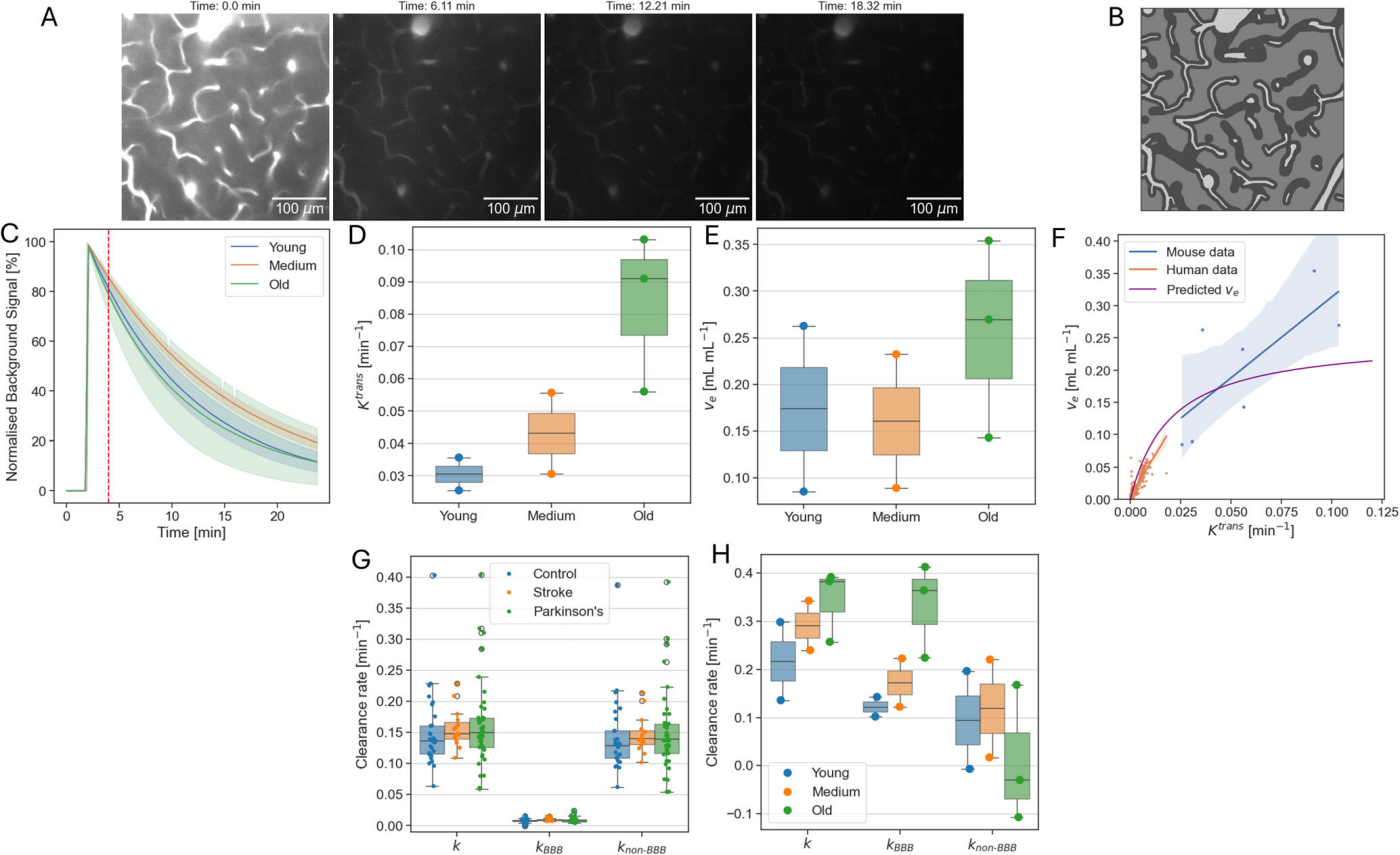
Results of Sulphorhodamine‐101 two‐photon microscopy in mice. (A) Plot of a signal time course for an example 87 week old mouse; *t* = 0 s relates to 2 min post injection. The tracer can be seen permeating the imaged region at *t* = 0 s and clearing with time. (B) Example segmentation of vessels (white), extravascular space (light gray) and excluded region (dark gray). Regions of the field of view were excluded from analysis if a vessel was found to be poorly resolved. (C) Normalized extravascular signal time course shows the quantitative drop in signal observed in Panel A. (D) Estimates of *K* ^trans^ obtained by fitting the extended Tofts model to data in Panel C. (E) Estimates of the interstitial volume fraction, *v*_e_ are closer to the literature estimates than human DCE‐MRI estimates, with data from the oldest mice being within the expected literature range (i.e., 25%–30% for anesthetized mice). (F) Linear regression analysis of the relationship between *K* ^trans^ and *v*_e_ for human and mouse data. Included in the plot is a prediction of the dependence of estimated *v*_e_ on *K* ^trans^ assuming a total clearance rate *k* = 0.15 min^−1^ and true *v*_e_ of 0.25. (G, H) Clearance rate estimates for human and mouse data, respectively, assuming *v*_e_ = 0.25. *k* = *k*_BBB_ + *k*_non‐BBB_.

Figure 4 shows results from the 2‐photon experiments. Figure 4A shows the parenchymal signal time course of SR‐101 between 2 and 22 min post‐injection. The signal behavior was consistent between animals; SR101 was observed to rapidly leak from vessels into parenchyma within 2 min, then rapidly clear from both vessels and parenchyma over the next 20 min. Figure 4B shows the vessel and background segmentation for an example two‐photon image. Figure 4C shows the extravascular signal for young, intermediate and old mice. The signal from before the start of the scan, denoted by a dashed red line, was extrapolated to the point of injection, assuming an exponential decay. The older mice were observed to have a faster clearance of SR101 than young and intermediate mice. Overall, this data supports the notion that in healthy appearing cortex, extravasated tracer is rapidly cleared from the brain, and does not accumulate as described by the Patlak model.

Figure 4D,E show the estimated *K* ^trans^ and *v*_e_ for SR101, under the assumption that clearance is entirely via the BBB (i.e., the extended Tofts model assumption). A trend to increase in *K* ^trans^ was observed with age, but this was not statistically significant. The absolute values of *K* ^trans^ were approximately 10–100 times larger than Gd‐DOTA as measured using DCE‐MRI. Estimates of *v*_e_ were higher than estimated with DCE‐MRI and more comparable to literature values. Figure 4F shows a linear regression analysis of the human DCE‐MRI and mouse two‐photon *v*_e_ estimates against *K* ^trans^. *K* ^trans^ and *v*_e_ for both Gd‐DOTA and SR101 were positively correlated and increase toward literature values for *v*_e_ as *K* ^trans^ increases. This reflects the fact that as *K* ^trans^ increases, so does BBB clearance. Thus, the extended Tofts assumption that clearance is only via the BBB becomes a more accurate representation of the underlying physiology, and *v*_e_ estimates become less biased. Figure 4G,H show the estimates of the parenchymal clearance rate *k* and the estimated contributions of clearance across the BBB (*k*_BBB_) and non‐BBB routes (*k*_non‐BBB_), assuming *v*_e_ = 25%. In that case, the human DCE‐MRI data shows a small contribution to clearance from the BBB (*k*_BBB_ = 0.009 ± 0.004 [min^−1^]) and an approximately 10 times larger contribution from non‐BBB pathways (*k*_non‐BBB_ = 0.1 ± 0.1 [min^−1^]). In the mouse 2P data, contribution from BBB and non‐BBB clearance are similar for young and middle‐aged mice (*k*_BBB_ = 0.12 ± 0.02 min^−1^ and *k*_non‐BBB_ = 0.1 ± 0.1 min^−1^, *p* = 0.96; *k*_BBB_ = 0.17 ± 0.05 min^−1^ and *k*_non‐BBB_ = 0.1 ± 0.1 min^−1^, *p* = 0.87, respectively), but with a significant difference in contributions for old mice (*k*_BBB_ = 0.36 ± 0.08 min^−1^ and *k*_non‐BBB_ = −0.03 ± 0.1 min^−1^, *p* < 0.01). The BBB clearance of SR101 in mice was predicted to be approximately 10 times faster than the BBB clearance of Gd‐DOTA in humans, whereas the non‐BBB clearance agreed well between humans and young and middle‐aged mice. The total clearance rate *k* was found to be approximately three times higher for SR101 in mice than for Gd‐DOTA in humans, mostly because of the enhanced BBB contribution. Under the assumption that *v*_e_ ∼25%, the clearance rate estimated from Gd‐DOTA DCE‐MRI appears to be primarily driven by non‐BBB mechanisms.

## 4 Discussion

DCE‐MRI is increasingly used to assess BBB leakage and glymphatic function in vivo. Gadolinium‐based contrast agents are either injected into the bloodstream and tracked as they cross the BBB or injected into the CSF compartment and tracked as they enter the parenchyma via perivascular spaces. In both cases, contrast agents that enter the extravascular extracellular space of the parenchyma eventually leave, either via re‐entry to the bloodstream or via non‐BBB clearance into CSF.

Estimating clearance from intravenous DCE‐MRI data is often discounted due to the widespread assumption that contrast agent efflux is too low to detect reliably. As such, the Patlak model, which assumes zero clearance, is typically used to extract estimates of *K* ^trans^. Here we show that when analyzing high temporal resolution DCE‐MRI data at an ROI level, models that describe finite clearance provide an improved fit compared to uptake only models, and that reliable estimates of Gd‐DOTA clearance rates can be obtained. Interestingly, when clearance was modeled, differences in *K* ^trans^ between controls and patients were observed, which were not evident in Patlak estimates of *K* ^trans^. This result suggests that subtle changes in BBB dysfunction may be missed when clearance of tracer from the brain is ignored.

While we found that modeling clearance improves fit quality, we also observed that estimates of *v*_e_ obtained using the extended Tofts model were biased relative to literature values [29]. This discrepancy between estimated and literature *v*_e_ was reduced as *K* ^trans^ increased, indicating the assumption that contrast agent clears solely into blood may be inaccurate at low *K* ^trans^, but becomes more acceptable as BBB permeability increases. This behavior is consistent with the presence of a secondary clearance pathway independent of the BBB (here termed non‐BBB clearance) which causes biases in extended Tofts estimates of *v*_e_ at low *K* ^trans^. Similar observations of lower‐than‐literature *v*_e_ were made by Barnes et al. [21] using a two‐compartment exchange model, but a convincing explanation for the bias was not given.

Surprisingly, we did not observe any disease‐specific difference in *k*, *k*_BBB_ or *k*_non‐BBB_ in humans despite detecting differences in *K* ^trans^ between groups. Literature evidence for alterations to brain clearance in these conditions is limited. A small number of studies have found lower DTI‐ALPS index in both Parkinson's Disease [39, 40] and post‐stroke [41], however the relation of this metric to brain clearance is contested [42, 43]. Having a more permeable BBB may not necessarily translate to increased clearance, particularly if the interstitial volume also changes. BBB clearance is inversely proportional to *v*_e_, thus if *v*_e_ increases it can compensate for increased *K* ^trans^ to maintain a constant BBB clearance. This effect is entirely consistent with vasogenic oedema that typically occurs following BBB breakdown. As observed in this study, extended Tofts model estimates of *v*_e_ are biased, which we have proposed is caused by incorrect modeling of the contrast agent clearance process. Therefore, it is highly possible that estimates of k, and the derived parameter *k*_non‐BBB_ obtained in this study are biased in a disease‐dependent manner, masking potential disease‐specific differences in clearance. Future work should aim to resolve this issue by refining how the non‐BBB clearance process is modeled, incorporating its dependence on the interstitial volume fraction and tortuosity of the interstitial space.

The contrast agent clearance rate measured in our studies (*k* ∼0.1 min^−1^) was five times faster than clearance of ^125^I‐Aβ_1‐40_ following direct brain injection as reported by Iliff et al. [12] (*k* ∼0.019 min^−1^) and about 30 times faster than clearance of ^125^I‐Aβ_1‐40_ measured by Shibata et al. [15] (*k* = 0.0029 ± 0.0002 min^−1^). Our rates were also five times faster than measurements of contrast agent clearance from the brain obtained in rats through compartmental modeling of Gd‐DTPA injected into the CSF [44]. The discrepancy in observed clearance rates between our data and that of others could be due to differences in the tracer size and BBB permeability of the tracers but could also be due to the invasive nature of direct brain and CSF injections used by others. Insertion of needles into the brain has been shown to transiently suppress influx and clearance of CSF tracers [45]. Another explanation may be that clearance rates depend on the delivery method of the tracer. It is known that non‐BBB clearance relies on a combination of diffusion (and possibly advection) of tracers through the extracellular space toward perivenous channels, where they are rapidly removed via advection. Thus, if the source of tracer is on average closer to perivenous channels for a given delivery method, then the time for molecules to diffuse toward the perivenous sinks will be shorter and thus tracers will clear faster. CSF tracer will enter the brain via peri‐arteriolar channels and will on average travel approximately 200 μm to the nearest perivenous channel [46]. If tracer is delivered via the capillaries, which are distributed approximated every 40–50 μm, then the average diffusion distance to the nearest perivenous channel will be 100 μm. This difference in diffusion distance corresponds to a factor of 4 in diffusion time and could partially explain why our measurements of clearance are five times faster than for other methods. This is supported by work by van Veluw et al. [47], who developed an innovative approach to deliver intravenous fluorescent dextran tracers into the extracellular space of brain tissue via vessel specific laser ablation. Fitting a mono‐exponential decay to their reported datapoints we obtain a clearance rate ∼0.08 min^−1^ which is similar to our observation of *k*_non‐BBB_ ∼0.1 min^−1^. The same study also found similar clearance rates for intravenously injected unconjugated fluorescein, which was found to readily extravasate under physiological conditions and rapidly clear from the brain, similar to our observation with SR101 [47]. Additionally, there is recent evidence of BBB‐impermeable europium‐DTPA fluorescent tracer being cleared in three phases following intra‐striatal injection with the fastest phase occurring possibly via pores in venules at rates comparable to our observations (*k* > 0.2 min^−1^) [48].

Since we have not measured the non‐BBB component of clearance directly, we do not speculate as to the specific route taken by Gd‐DOTA or SR101, only that the existence of an unaccounted for and significant component of clearance via non‐BBB routes could explain the bias in estimates of *v*_e_ observed with the extended‐Tofts model. Shibata et al. [15] reported a similar distinction between BBB and non‐BBB clearance when measuring efflux of ^125^I‐Aβ_1‐40_; in their case, non‐BBB clearance was interpreted as occurring through ISF bulk flow. While bulk flow of solutes and tracers within the interstitial space has been debated and studied for over 50 years [7, 8, 9, 10, 11, 49, 50, 51], there are several more recent hypotheses relating to how solutes are removed from the interstitial space, namely the intramural peri‐arterial drainage (IPAD, [52]) and perivenous drainage via the glymphatic system [12]. Recent concern into Gd‐DOTA accumulation in the brain has led to identification of Gd‐DOTA deposits in the brain following repeated injections, particularly near blood vessels [53, 54, 55, 56], supporting both drainage theories. It is also possible that very low estimates of *v*_e_ are a result of Gd‐DOTA accumulating in the perivascular spaces. Unfortunately, we were unable to test this hypothesis due to lack of a suitable approach to measure Gd‐DOTA in vivo or in brain samples at the required spatial resolution. SR‐101 2‐photon microscopy acquired in mouse brain did not show evidence of perivascular accumulation (Figure S6).

Our study compared human DCE‐MRI data with a novel approach to measuring the kinetic behavior of small tracers in mouse brain with an intact dura. Since it is not possible to detect Gd‐DOTA via optical approaches, we used a readily available fluorescent dye of similar molecular weight to Gd‐DOTA (MW 558.64 vs. MW 606.71 g/mol, respectively). SR101 was found to leak far more readily into mouse brain than Gd‐DOTA in humans, and *v*_e_ estimates agreed more favorably with literature data. A positive correlation between *K* ^trans^ and *v*_e_ was found, similar to our human measurements, further supporting our hypothesis that bias in extended Tofts estimates of *v*_e_ is driven by the presence of a secondary clearance process. A potential limitation of using SR101 for the purpose of *v*_e_ estimation is its known affinity to astrocytes [57]. We found some progressive staining of cells with SR101, but this contributed insignificantly to the background signal within the 20‐min scan duration (Figure S7). Therefore, we are confident that our estimates of *v*_e_ obtained using SR101 reflect the interstitial compartment only. DCE‐MRI and two‐photon microscopy have vastly different spatial resolution and coverage. The imaging volume of the microscopy data was about 4000 times smaller than the volume of a single voxel in the DCE‐MRI study. The microscopy data would therefore likely be far more subject to tissue heterogeneity, which could explain the greater level of variation in *K* ^trans^ and *v*_e_ estimates compared to DCE‐MRI data.

Several acquisition or modeling factors may cause a clearance model to fit data better than an uptake model, even if clearance is zero. These include *T*_1_ and *T*_2_/*T*_2_* effects, incorrect assumptions about instantaneous mixing, incorrect modeling of water exchange, and scanner drift. Interstitial fluid and CSF have different *T*_1_ and *T*_2_ values and could produce different signal changes in response to contrast agent. In our DCE‐MRI protocol, we use a very short *TE*, so *T*_2_ effects should be negligible. However, the *T*_1_‐weighting of the DCE‐MRI scan means that the much longer *T*_1_ of CSF could affect the validity of the signal to concentration conversion, if contrast agent enters a CSF filled sub‐voxel compartment. To address this, we simulated the effect of GBCA concentration on the spoiled gradient echo signal (Figure S8) for a pure tissue ROI and a ROI containing tissue and CSF. We found that signal changes caused by contrast agent within these two ROIs are almost identical; therefore, from an acquisition and modeling perspective, it does not matter whether contrast clears from the ROI via perivascular spaces or not. Assumptions about instantaneous mixing and dispersion were found to not have a major effect on fit quality, as evidenced by a large difference in AIC when compared to the extended Tofts model. Finite BBB water exchange was the most influential factor; however, extended Tofts model fits were still superior. While modeling finite BBB water exchange provides a plausible alternative to clearance, two‐photon data of SR101 leakage, which is not confounded by water exchange, supports the notion that in healthy appearing brain tissue, extravasated tracer is rapidly cleared from the brain and does not accumulate, as described by the Patlak model. Our analysis of signal drift also strongly indicates that linear or exponential drift in estimated concentrations cannot explain why the extended Tofts model is preferred over Patlak. The temporal resolution of data appears to be quite important for detecting the effect of clearance (Figure S4). Given the slow kinetics of clearance, we hypothesize this increased sensitivity is caused by the availability of more data points, increasing the effective SNR, rather than increased sampling rate.

Our estimates of BBB and non‐BBB tracer clearance are based on a literature value of *v*_e_ [29]. There are numerous noninvasive approaches to *v*_e_ quantification that may be used to estimate *v*_e_, potentially allowing *k*_BBB_ and *k*_non‐BBB_ to be separated without assumptions. Sodium MRI can be used to quantify *v*_e_ based on the known intra‐ and extracellular distribution of sodium ions [58, 59]. Soma and Neurite Density MRI (SANDI) can be used to extract the extra‐cellular space in addition to modeling the intra‐neurite and intra‐soma spaces from diffusion MRI with *v*_e_ estimates of 20%–35% in GM [60, 61].

In conclusion, our data show that high temporal resolution DCE‐MRI acquisitions can detect clearance of Gd‐DOTA from the human brain. Large biases in *v*_e_ were found, which we hypothesize are caused by the presence of significant and dominant non‐BBB contribution to tracer clearance in the context of individuals with subtle BBB impairment. Clearance rates measured using both DCE‐MRI in humans and two‐photon imaging of SR101 in mice agreed well with estimates obtained using intravascular delivery methods but were of the order 5–10 times faster than invasive methods that rely on direct brain or CSF injections. Modeling clearance likely requires higher data quality compared to Patlak modeling (higher SNR and temporal resolution), although the specific requirements are currently undefined and should be explored in future work. Identifying the routes of Gd‐DOTA clearance from the brain under different BBB leakage conditions should also be of high importance for future investigation. In summary, this study highlights the potential of intravenous DCE‐MRI to be used as a noninvasive method to assess brain clearance mechanisms.

## Funding

This work was supported by the Sydney Driscoll Neuroscience Foundation and the Engineering and Physical Sciences Research Council, EP/M005909/1.

## Conflicts of Interest

Geoff J. M. Parker has multiple conflicts of interest to declare: G.J.M.P. receives salary from, is a shareholder in, and is a director of Bioxydyn Limited, a company with an interest in imaging biomarkers. G.J.M.P. is a shareholder in, and is a director of Queen Square Analytics Limited, a company with an interest in imaging biomarkers. G.J.M.P. is a shareholder in, and is a director of Quantitative Imaging Limited, a company with an interest in imaging biomarkers. G.J.M.P. receives research funding from Eli Lilly and Company. The other authors declare no conflicts of interest.

## Supporting information


**Figure S1:** Following the dextran injection (seen as green), an exploratory image of the entire cranial window was taken to locate candidate regions for the SR‐101 scan. The square box was chosen for further imaging.
**Figure S2:** Data showing the effects of excluding early datapoints on ΔAIC calculated from extended Tofts and Patlak fits. The extended Tofts model provides significantly lower AIC (negative ΔAIC) for all time windows tested. The fit quality between extended Tofts and Patlak becomes more similar as more data is excluded.
**Figure S3:** Results of DCE‐MRI kinetic modeling in white matter of the human brain. (A, B) Example plots of the Patlak and extended Tofts models for an example healthy control subject after removing the first 100 s of data. (C) The difference in Akaike information criterion values between Patlak and extended Tofts models for the three patient categories (D) The estimates of the tracer transfer constant *K* ^trans^ using the Patlak and extended Tofts models for the three patient groups. The estimates are significantly higher for the extended Tofts model (*p* < 0.0001). (D) Estimates of the interstitial volume fraction *v*_e_ from the extended Tofts model. (E) Estimates of the clearance rate *k* from the extended Tofts model.
**Figure S4:** Results of DCE‐MRI kinetic modeling in gray and white matter in data that has been downsampled to 73 s per volume, the temporal resolution of Heye et al. [6] ( https://doi.org/10.1016/j.neuroimage.2015.10.018). It can be clearly observed that the difference in AIC between extended Tofts and Patlak is smaller than data sampled at the original resolution (see Figure S2).
**Figure S5:** Effects of scanner drift on model selection. (A) Measured signal drift assuming a linear model. (B, C) Synthetic data simulated assuming an exponential drift model with decreasing concentration (drift down) and increasing concentration (drift up). (D) AIC difference plot of extended Tofts versus Patlak. The AIC difference plot has a mean difference of approximately +2, indicating preference for the Patlak model.
**Figure S6:** Sulphorhodamine‐101 2‐photon data collected to test the hypothesis of perivascular tracer accumulation. Perivascular accumulation of tracer is a plausible explanation for the low *v*_e_ estimates obtained during extended Tofts model fitting and would be consistent with a gradual dilation of vessel diameter during the scan period. (A) Microscopy image showing placement of line profiles. (B) Full width half maximum of the profile normalized to peak amplitude. (C) The full width half maximum of each mouse group as a function of time, showing no change in vessel diameter.
**Figure S7:** Data showing Sulphorhodamine‐101 uptake and clearance in a representative mouse from the older group. (A) The image shows SR‐101 signal 2 min after injection. Signal is predominantly in vessels, but substantial signal can also be seen in background regions, indicating leakage has occurred. (B) The same image acquired at *t* = 20 min post injection, showing reduced signal in background, and uptake in small nuclear structures, likely reflecting cellular uptake. (C) Cellular structures were segmented and the volume fraction calculated as a function of time (D) Signal intensity in cellular signal relative to total background showing that cellular uptake accounts for a very small fraction of the overall signal.
**Figure S8:** Simulations of SPGR signal changes due to changing gadolinium based contrast agent (GBCA) in a region of interest (ROI) containing pure tissue or tissue and cerebrospinal fluid (CSF). The simulated acquisition parameters were set to be the same as in the acquired patient data. Tissue was simulated with a *T*_1_ of 1.5 s and CSF with a *T*_1_ of 4 s. *T*_2_ effects were assumed to be negligible due to use of a very short TE. In the ROI containing CSF, CSF was assumed to occupy 5% of the total ROI volume. The absolute signal shows differences between the pure tissue and tissue + CSF ROIs, but the signal difference is extremely small for typical tissue contrast agent concentrations. As such, presence of contrast agent within sub‐ROI CSF cannot be distinguished from its presence within tissue water.

## Data Availability

Raw data used within this study are available upon reasonable request from the corresponding author. Derived data and analysis scripts can be found on https://github.com/nimo‐group/DCE‐MRI‐brain‐clearance‐2025.
