# Supplementary material for: Brain Clearance of Contrast Agent in Intravenous DCE‐MRI Is Measurable and Cannot Be Explained by Clearance Across the BBB Alone
Source: Magn Reson Med. 2026 Jul 18;96(5):2309–20. doi: 10.1002/mrm.70514 (PMC13527286; doi:10.1002/mrm.70514)
Supplement: Supplementary file 1 [file MRM-96-2309-s001.docx]

## Supplementary material

**Figure S1.** Following the dextran injection (seen as green), an exploratory image of the entire cranial window was taken to locate candidate regions for the SR-101 scan. The square box was chosen for further imaging.

**Figure S2**. Data showing the effects of excluding early datapoints on ΔAIC calculated from extended Tofts and Patlak fits. The extended Tofts model provides significantly lower AIC (negative ΔAIC) for all time windows tested. The fit quality between extended Tofts and Patlak becomes more similar as more data is excluded.

Figure S3. Results of DCE-MRI kinetic modelling in white matter of the human brain. A-B) Example plots of the Patlak and extended Tofts models for an example healthy control subject after removing the first 100 seconds of data. C) The difference in Akaike information criterion values between Patlak and extended Tofts models for the three patient categories D) The estimates of the tracer transfer constant *K*^trans^ using the Patlak and extended Tofts models for the three patient groups. The estimates are significantly higher for the extended Tofts model (p<0.0001). D) Estimates of the interstitial volume fraction *v*_e_ from the extended Tofts model. E) Estimates of the clearance rate *k* from the extended Tofts model.

**Figure S4.** Results of DCE-MRI kinetic modelling in gray and white matter in data that has been downsampled to 73s per volume, the temporal resolution of Heye et al. 2016 (<https://doi.org/10.1016/j.neuroimage.2015.10.018>). It can be clearly observed that the difference in AIC between extended Tofts and Patlak is smaller than data sampled at the original resolution (see Supplementary Figure 2)**.**

Figure S5. Effects of scanner drift on model selection. A) Measured signal drift assuming a linear model. B-C) Synthetic data simulated assuming an exponential drift model with decreasing concentration (drift down) and increasing concentration (drift up). D) AIC difference plot of extended Tofts vs Patlak. The AIC difference plot has a mean difference of approximately +2, indicating preference for the Patlak model.

Figure S6. Sulphorhodamine-101 2-photon data collected to test the hypothesis of perivascular tracer accumulation. Perivascular accumulation of tracer is a plausible explanation for the low v_e_ estimates obtained during extended Tofts model fitting and would be consistent with a gradual dilation of vessel diameter during the scan period. A) Microscopy image showing placement of line profiles. B) Full width half maximum of the profile normalised to peak amplitude. C) The full width half maximum of each mouse group as a function of time, showing no change in vessel diameter.

Figure S7. Data showing Sulphorhodamine-101 uptake and clearance in a representative mouse from the older group. A) The image shows SR-101 signal two minutes after injection. Signal is predominantly in vessels, but substantial signal can also be seen in background regions, indicating leakage has occurred. B) The same image acquired at t = 20 minutes post injection, showing reduced signal in background, and uptake in small nuclear structures, likely reflecting cellular uptake. C) Cellular structures were segmented and the volume fraction calculated as a function of time D) Signal intensity in cellular signal relative to total background showing that cellular uptake accounts for a very small fraction of the overall signal.

**Figure S8.** Simulations of SPGR signal changes due to changing gadolinium based contrast agent (GBCA) in a region of interest (ROI) containing pure tissue or tissue and cerebrospinal fluid (CSF). The simulated acquisition parameters were set to be the same as in the acquired patient data. Tissue was simulated with a *T*_1_ of 1.5s and CSF with a *T*_1_ of 4s. *T*_2_ effects were assumed to be negligible due to use of a very short TE. In the ROI containing CSF, CSF was assumed to occupy 5% of the total ROI volume. The absolute signal shows differences between the pure tissue and tissue + CSF ROIs, but the signal difference is extremely small for typical tissue contrast agent concentrations. As such, presence of contrast agent within sub-ROI CSF cannot be distinguished from its presence within tissue water.
